# Mesenchymal stem cells desensitize castration-resistant prostate cancer to docetaxel chemotherapy via inducing TGF-β1-mediated cell autophagy

**DOI:** 10.1186/s13578-020-00494-0

**Published:** 2021-01-07

**Authors:** Yang Yu, Fu-han Yang, Wen-tao Zhang, Ya-dong Guo, Lin Ye, Xu-dong Yao

**Affiliations:** Department of Urology, Shanghai Tenth People’s Hospital, Tongji University School of Medicine, Shanghai, 200072 China

**Keywords:** Mesenchymal stem cells, Prostate cancer, Docetaxel resistance, Autophagy, Transforming growth factor-β1

## Abstract

**Background:**

Mesenchymal stem cells (MSCs) have been proved to drive castration resistant prostate cancer (CRPC). In this study, we aim to investigate the contribution of MSCs to the development of docetaxel resistance in CRPC cells and its potential mechanisms.

**Methods:**

The effect of MSCs on CRPC cells resistance to docetaxel was determined using in vivo and in vitro approaches. CCK8 and PI/Annexin V-FITC assay were used to examined the cell viability and apoptosis. The concentration of transforming growth factor-β1 was measured by enzyme-linked immunosorbent assay and small interfering RNA was used for functional analyses.

**Results:**

MSCs significantly reduced the sensitivity of CRPC cells to docetaxel-induced proliferation inhibition and apoptosis promotion in vivo and in vitro. CRPC cells cocultured with MSCs under docetaxel administration have an increased autophagy activation, while autophagy inhibitor could effectively reversed MSCs-induced resistance to docetaxel. Additionally, MSCs-induced CRPC cell autophagy increase under docetaxel administration depends on MSCs secreting TGF-β1 and inhibition of TGF-β1 secretion in MSCs could consequently increase the sensitivity of CRPC cells to docetaxel.

**Conclusions:**

These results suggest that docetaxel administrated CRPC cells may elicit MSCs secreting TGF-β1 increase, which desensitizes CRPC to docetaxel chemotherapy accelerating chemoresistance occurrence via inducing cell autophagy.

## Introduction

Prostate cancer (PCa) is the most common cancer and the second leading cause of cancer-related mortality among men in the United States [1]. In China, PCa shows an increasing incidence and mortality in recent years [2]. Early staged and localized PCa can be well controlled by prostatectomy or radiotherapy. For locally advanced and metastatic PCa, androgen deprivation therapy (ADT) is currently considered as the most effective treatment, giving a 70% initial effective rate [3]. However, almost all of them with initially castration-sensitive prostate cancer (CSPC) would eventually develop into castration-resistant prostate cancer (CRPC) after a period of ADT. Currently, docetaxel-mediated antimitotic chemotherapy is typically used as the first-line standard treatment in metastatic CRPC patients [4]. However, it only gives a moderate survival advantage as patients eventually acquire resistance resulting in therapeutic failure. Clarifying the mechanism of docetaxel chemoresistance in CRPC can provide a basis for new treatment approaches.

Resistance to docetaxel attributes to numerous different mechanisms and many of them are related to abnormal molecular regulation, which involved in cell survival and death [5]. Recently, increasing evidence suggests that tumor microenvironment (TME) may play a key role in occurrence of docetaxel resistance [6]. TME is comprised of tumor epithelial cells and diverse non-malignant stromal cell types. Mesenchymal stem cells (MSCs), as a heterogeneous subset of stromal stem cells, are an important component of TME. MSCs originate from the mesodermal germ layer and main exist in bone marrow [7]. MSCs can migrate into PCa tumor sites and perform critical roles ranging from supporting tumor cell proliferation to inducing tumor cell metastasis, accelerating tumor development [8–10]. Besides that, numerous data from clinical studies have shown a strong association between MSCs density and poor prognosis in various types of human cancer [11]. MSCs have been also reported to be used as a predictor of reduced cancer-free survival interval in PCa [12]. Our previous studies have demonstrated that MSCs could promote androgen-dependent PCa cells form a tumor xenograft in castrated mice, suggesting that MSCs could promote PCa cells growth from androgen-dependent into androgen-independent manner and contribute to PCa castration resistance progression [13]. However, the role of MSCs in the development of CRPC to docetaxel chemoresistance remains unclear.

In the present study, we used human CRPC cell lines PC3 and DU145 to investigate the effect of MSCs on the occurrence of chemoresistance to docetaxel and explore the potential mechanisms. The results indicate that MSCs could desensitize CRPC cells to docetaxel chemotherapy and contribute to chemoresistance via inducing TGF-β1-mediated cell autophagy.

## Materials and methods

### Cell lines and culture

Human prostatic carcinoma cell lines, including PC3, DU145 cells, and bone marrow-derived MSCs were purchased from the Cell Bank of Type Culture Collection of Chinese Academy of Sciences, Shanghai Institute of Cell Biology, Chinese Academy of Sciences. PC3, DU145 cells were cultured in RPMI-1640 medium supplemented with 10% fetal bovine serum (FBS). MSCs were cultured in MSCs basal medium (all from Invitrogen, Carlsbad, CA, USA). MSCs were transfected with the adenoviral vector GFP-mock (Invitrogen). After transfection about 48 h, MSCs-GFP were collected for further experiments. All cells were cultured at 37 °C in a 5% CO_2_ humidified atmosphere.

### In vivo xenograft experiment

Nude mice, 6–8 weeks old, were obtained from the Shanghai Experimental Animal Center of the Chinese Academy of Sciences, Shanghai, China, and housed in pathogen-free conditions. All aspects of the animal care and experimental procedures were in accordance with the Guide for the Care and Use of Laboratory Animals and approved by the Chinese Academy of Sciences’ Committee on Animals. PCa cells were prepared as single-cell type suspensions (1 × 10^6^ cells in 200 μL PBS) and subcutaneously administrated in the armpit area of nude mice. When tumors grew to approximate 200 mm^3^ size, nude mice were randomly divided into three groups: with solvent or docetaxel treatment (15 mg/kg/week via intraperitoneal injection, 5 mice per group). With that, mice were injected with the green fluorescent protein (GFP)-labeled MSCs (MSCs-GFP) through tail vein every 3 days. Mice were examined every day and tumor growth was evaluated by measuring the length and width of tumor mass. All tumor-bearing mice survived until they were sacrificed at the end of the experiment, then tumors were removed and dissected quickly for frozen section preparation, while others were stored at − 80 °C.

### Cell apoptosis assay

Cells (2 × 10^5^ cells/well) were cultured in 6-well plates to 70–80% confluence. The cells were then treated with docetaxel for 48 h. PI/Annexin V-FITC assay was used to measure apoptotic cells by flow cytometry according to the manufacturer’s instruction (Keygen Biotech. Co., Ltd, Nanjing, China, Cat.KGA108). The cells were collected by trypsinization and were washed with ice cold phosphate buffered saline (PBS). Cells were then incubated in 300 μL of 1 × binding buffer containing 5 μL Annexin V and 5 μL PI for 30 min at room temperature in the dark. Apoptosis of cells was measured on a BD FACScan flow cytometer (BD Biosciences). At least 30,000 gated events were acquired from each sample. Results are expressed as the percentage of apoptotic cells (PI and Annexin V positive) in the gated cell population.

### Proliferation assays

Cells were plated at a density of 2 × 10^5^ cells per well in 6-well plates cocultured with MSCs and treated with docetaxel (20 µM) for 48 h. CCK8 test was performed to evaluate the extent of cell proliferation (OD values) according to the manufacturer’s instructions.

### Real-time quantitative PCR (RT-PCR)

To quantify mRNA expression of PCNA, caspase-3 and TGF-β1, total RNA was isolated using Trizol reagent (Invitrogen) and cDNA synthesis was performed using the Prime Script RT reagent Kit (Takara, Kyoto, Japan) according to the manufacturer’s specifications. Quantitative PCR was performed using SYBR Green PCR Kit (Applied Biosystems, Carlsbad, CA, USA) according to the manufacturer’s instructions. β-Actin was used as an internal control for RNA integrity and loading normalization.

### Western blot analysis

Cells were lysed in RIPA lysis buffer (Beyotime) with 1 mM PMSF. Equal amount of protein was separated by SDS-PAGE and transferred to NC membrane. The membranes were washed, blocked and incubated with specific primary anti-human antibodies against p62/SQSTM1 (all from Cell Signaling Technology, Inc., Danvers, MA, USA), LC3 (Novus Biologicals, Littleton, CO), TGF-β1 and β-actin (Abcam, Cambridge, MA, USA), followed by incubation with horseradish peroxidase-conjugated secondary antibodies (Hangzhou HuaAn Biotech). Signals were visualized by chemiluminescent detection (Beyotime).

### Enzyme linked immunosorbent assay (ELISA)

ELISA assays were performed using commercial ELISA kits (R&D Systems, Minneapolis, MN) according to manufacturer instructions. Assays were performed in duplicates, and readings were compared with standard curves obtained with standard protein provided with the kit. Means and standard deviations of concentrations in triplicate samples were compared by *t* test.

### RNA interference

Cells (1 × 10^6^) growing to 50–60% confluence in 10 cm petri dishes were transfected with TGF-β1 siRNA sequences (sense: 5′-CACUGCAAGUGGACAUCAATT-3′; antisense: 5′-UUGAUGUCCACUUGCAGUGTT-3′) or their corresponding mock sequences (sense: 5′-UUCUCCGAACGUGUCACGUTT-3′; antisense: 5′-ACGUGACACGUUCGGAGAATT-3′) using a Lipofectamine 2000 kit (Invitrogen, Cat.11668-019) with the procedure provided by the manufacturer. Cells were observed under a fluorescence microscope and harvested 48 h after transfection.

### Transient transfection

Fugene HD transfection reagent (Calbiochem, La Jolla, CA) was used to transfect cells with GFP-LC3 expressing plasmids according to the manufacturer’s instructions. After initial treatment, autophagy was detected by counting the number of GFP-LC3-positive dots per cell under fluorescence microscope (Olympus IX71).

### Electron microscopic analysis

Cells were fixed in 2.5% glutaraldehyde in PBS (pH 7.4) for 2 h at room temperature, then postfixed in 1% osmium tetroxide in water for 1 h, dehydrated in an ascending series of ethanol, and at last embedded in araldite (Basel, Switzerland). After solidified, 50 nm sections were cut on a LKB-I ultramicrotome and picked up on copper grids, post-stained with uranyl acetate and lead citrate, and observed in a Philips CM-120 TEM.

### Statistical analysis

All of the experiments were repeated at least three times. Final data were expressed as mean ± standard deviation (SD). Statistical analysis of the data was done by using GraphPad Prism 5. Student’s t-test was used to compare between mean values of two groups. Value of at least P < 0.05 was considered statistically significant.

## Results

### MSCs accelerate CRPC cells resistance to docetaxel

**Fig. 1:**
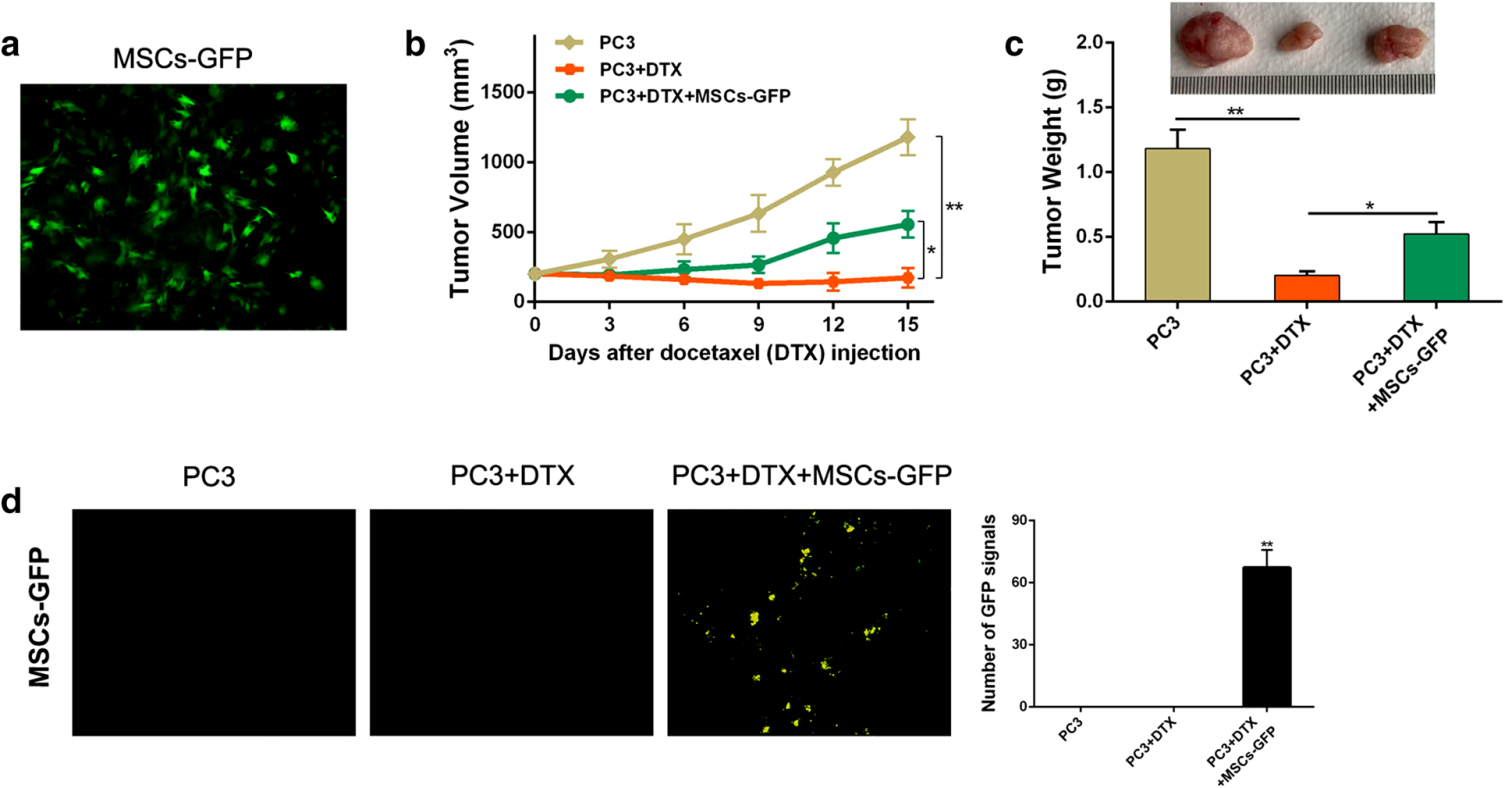
MSCs desensitize CRPC cells to docetaxel in vivo. **a** MSCs were transfected with the adenoviral vector GFP-mock (Invitrogen) to be marked. After transfection about 48 h, MSCs-GFP were detected by fluorescence microscope (original magnification ×200). **b** Mice with PC3 tumors were injected with MSCs-GFP or not through tail vein every 3 days, while mice were treated with docetaxel (DTX) or not. Tumor volume was observed and calculated using the formula: volume = width^2^ × length × 0.5236. **c** After docetaxel (DTX) injection for 15 days, tumor tissues were removed from mice (tumors from untreated MSCs-GFP mice as control) for the further experiments. Tumor weights were measured. **d** Tumor tissues were embedded in Tissue-Tek OCT compound and snap frozen in liquid nitrogen. Cryostat sections (6 mm thick) were prepared using a Leica CM1950 cryostat. GFP fluorescence signal was then analyzed with a fluorescence microscope (original magnification ×200). *P < 0.05; **P < 0.01

Firstly, we infected MSCs with an adenovirus vector to obtain GFP-labeled MSCs (Fig. 1a). Then studies were performed in PC3 xenograft mouse model. As shown in Fig. 1b, c, docetaxel could effectively inhibit prostate tumor growth. However, when MSCs-GFP were injected through nude mouse tail vein, the docetaxel-induced inhibition of PC3 cell growth was attenuated and the tumor would grow faster than before. The volume and weight of tumor were consequently both increase (Fig. 1b, c). To investigate whether MSCs could migrate into PCa sites, we also performed frozen sections to detected GFP signals in tumors. High numbers of GFP signals in frozen sections were detected in tumors removed from mice injected with MSCs-GFP (Fig. 1d). The results showed that MSCs desensitize CRPC cells to docetaxel accelerating chemoresistance in vivo.

### MSCs alleviate docetaxel-induced apoptosis in CRPC cells

**Fig. 2:**
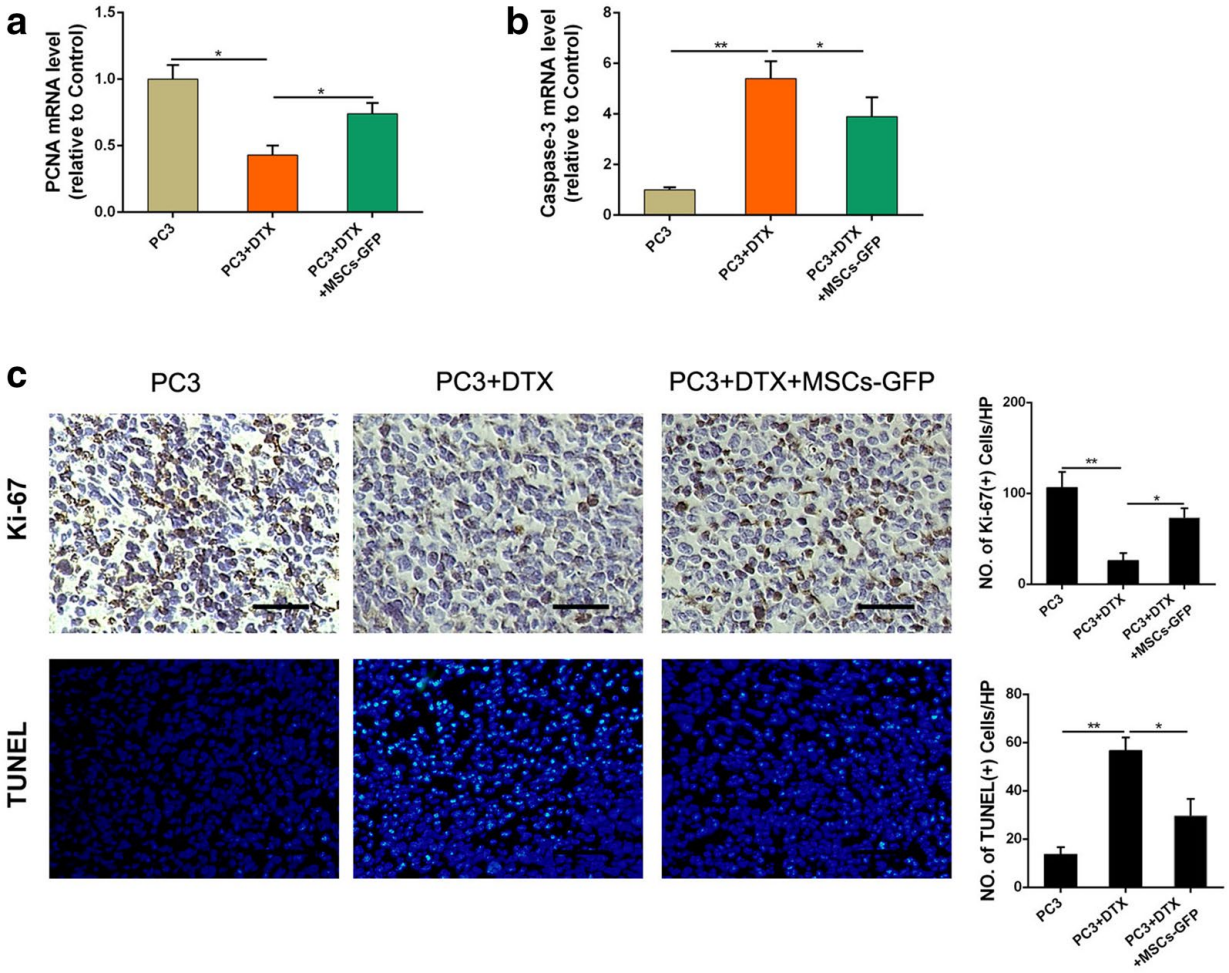
MSCs alleviate docetaxel-induced CRPC cells proliferation decrease and apoptosis increase in vivo. **a** Real-time PCR was employed to examine the PCNA expression level of tumors. Results were reported as ratio to control group. Real-time PCR was employed to examine the Caspase-3 expression level of tumors. Results were reported as ratio to control group. **b** Tumor tissues were analyzed for immunohistochemical staining of Ki-67. Apoptosis of 4T1 tumor tissues were determined by TUNEL assay. **c** Typical photographs were presented (original magnification ×200). The quantification of Ki-67-positive cells per HP and TUNEL-positive tumor cells was shown as mean ± SD. *P < 0.05; **P < 0.01

To evaluate the tumor cells proliferation and apoptosis induced by docetaxel, the mRNA expression of PCNA (a cell proliferation indicator) and Caspase-3 (a cell apoptosis indicator) were measured by real-time PCR. As shown in Fig. 2a, b, docetaxel treatment group induced a lower expression level of PCNA and a higher expression level of Caspase-3 than those of PC3 group. However, when MSCs-GFP were injected, the docetaxel-induced PCNA low expression and Caspase-3 high expression were significantly attenuated. We also analyzed tumor tissues sections with Ki67 and TUNEL, markers for proliferative and apoptotic response respectively. PC3 tumors in docetaxel treatment group showed a marked increase in number of Ki67-positive cells and an obvious decrease in number of TUNEL-positive cells when MSCs-GFP administrated (Fig. 2C).

**Fig. 3:**
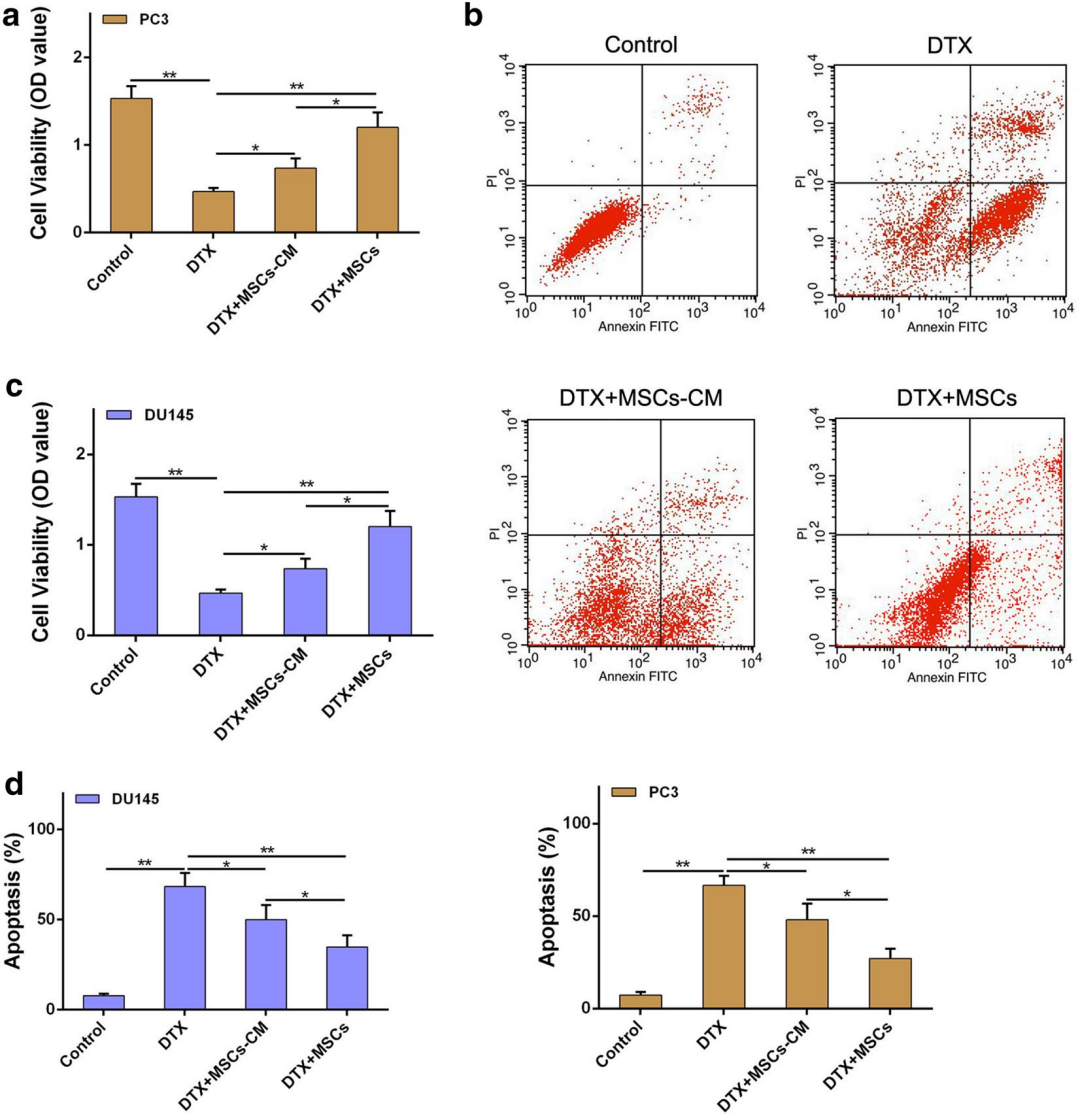
MSCs alleviate docetaxel-induced CRPC cells proliferation decrease and apoptosis increase in vitro. **a** PC3 cells (2 × 10^5^/well) were cocultured with MSCs in a 6-well plate or the conditioned medium collected from MSCs were added in PC3 culture medium with an existence of DTX (20 μM) for 48 h. CCK8 assay was used to examined the cell viability of PC3 cells. **b** The cell apoptosis of PC3 cells was analyzed by flow cytometry using Annexin V-FITC/PI. **c** CCK8 assay was used to examined the cell viability of DU145 cells. **d** The cell apoptosis of DU145 cells was analyzed by flow cytometry using Annexin V-FITC/PI. *P < 0.05; **P < 0.01

In addition, we also performed in vitro experiment using two human CRPC cell lines (PC3 and DU145) to verify the effect of MSCs on proliferation and apoptosis of CRPC cells administrated with docetaxel. As shown in Fig. 3a and c, CRPC cell proliferation was significantly inhibited by docetaxel, while MSCs could effectively activate CRPC cell proliferation in the presence of docetaxel. Correspondingly, MSCs also caused a significant reduction of docetaxel-induced apoptosis in CRPC cells (Fig. 3b, d). Together, these results indicate that MSCs could alleviate docetaxel-induced CRPC cell proliferation inhibition and apoptosis increase.

### MSCs promote CRPC cells docetaxel chemoresistance via inducing cell autophagy

**Fig. 4:**
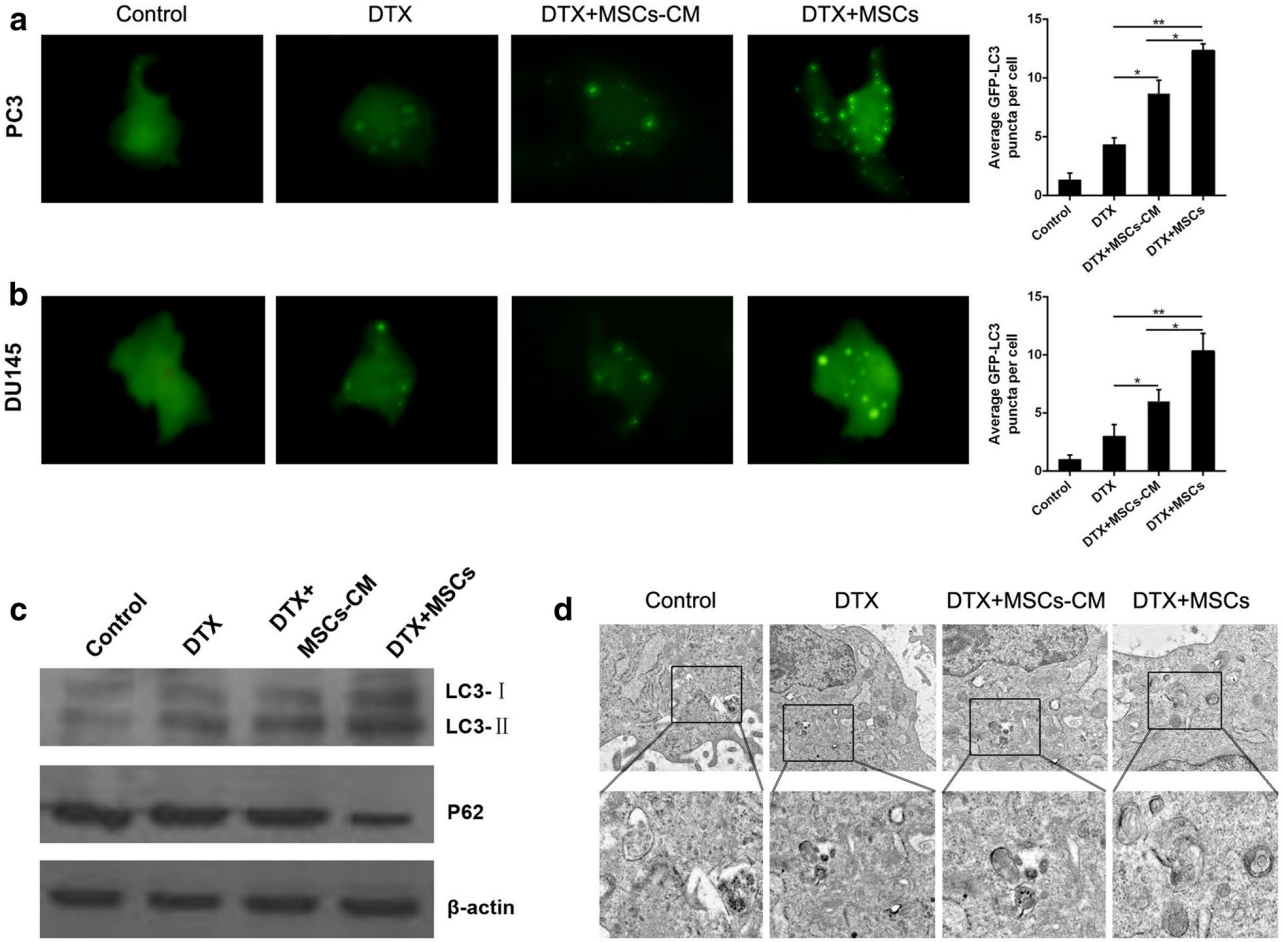
MSCs induce autophagy in CRPC cells. **a** GFP-tagged LC3 expression vector has been utilized to demonstrate the occurrence of autophagy in PC3 cells and was detected using an inverted fluorescence microscope. **b** GFP-tagged LC3 expression vector has been utilized to demonstrate the occurrence of autophagy in DU145 cells and was detected using an inverted fluorescence microscope. **c** Endogenous LC3 expression was detected by western blotting with β-actin as a loading control. The conjugated and unconjugated form of LC3 is referred to as LC3-II and LC3-I, respectively. The protein level of p62 was also analyzed by western blotting. **d** Representative electron micrographs (original magnification ×3000) clearly exhibited the autophagic vacuoles in the cytoplasm content. *P < 0.05; **P < 0.01

Several studies have reported that autophagy contributes to tumor cell resistance to chemotherapeutic agents [14]. We then explored if autophagy was involved in MSCs-induced docetaxel chemoresistance in CRPC cells. Firstly, we expressed GFP-LC3 fusion protein in PC3 and DU145 cells using an expression vector encoding GFP-LC3 which is concentrated in autophagic vacuoles within cells undergoing autophagy. As shown in Figure 4A and B, the number of punctate GFP significantly increased in PC3 and DU145 cells when coculturing with MSCs, indicating that MSCs could effectively induce autophagy in PC3 and DU145 cells. LC3-II, the conjugated form of LC3, is targeted to the autophagosome membrane and its accumulation means autophagy activation. We then performed western blotting to measure LC3-II level in PC3 cells. Results in Figure 4C showed that PC3 cells with docetaxel administration have higher expressions of LC3-II upon coculture with MSCs, indicating increased autophagy flux. We also examined another specific autophagy indicator P62 and obtained the consistent consequence. Meanwhile, electron microscopic analysis in Figure 4D showed the autophagosome formation in PC3 cells cocultured with MSCs under docetaxel administration.

**Fig. 5:**
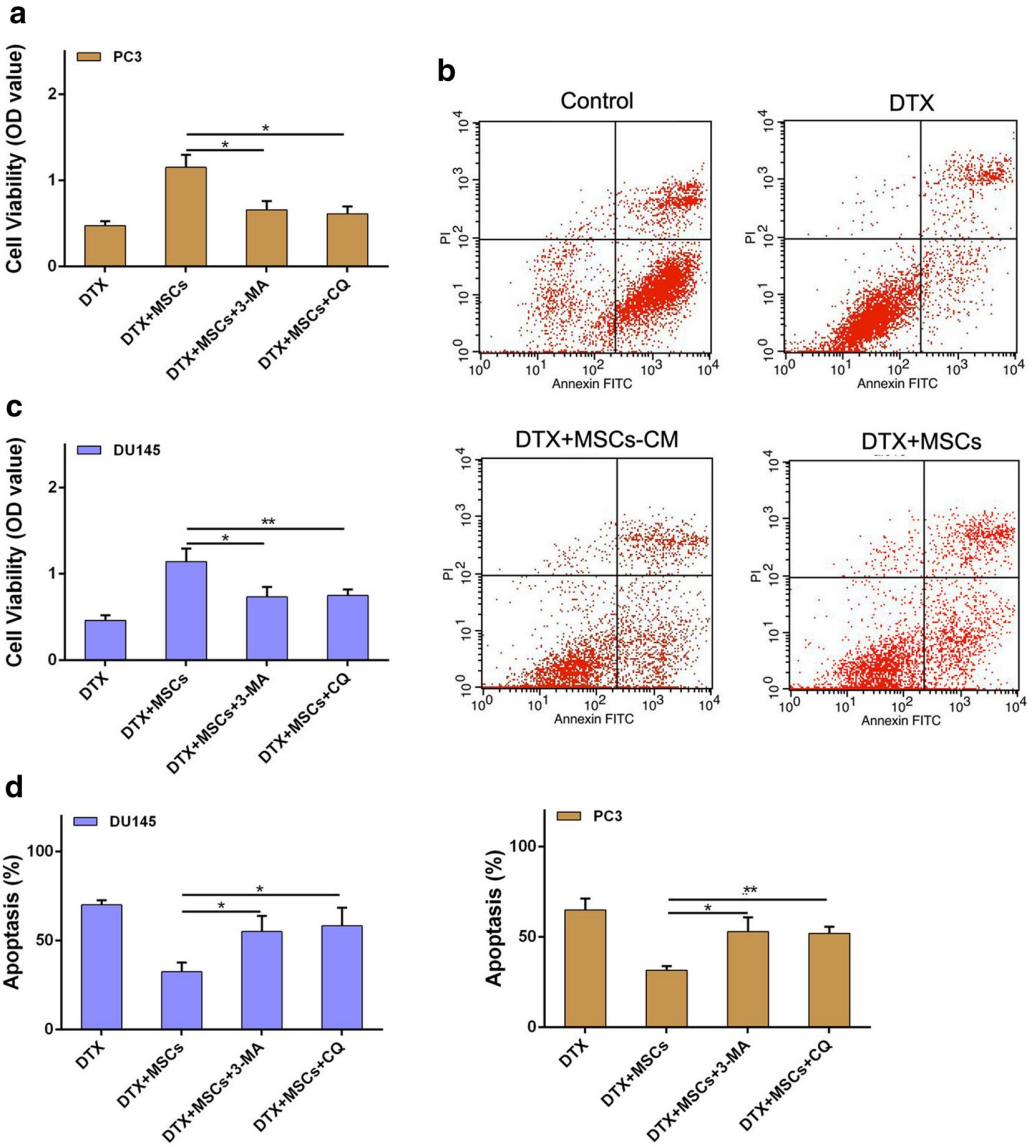
Inhibition of autophagy restored CRPC cell sensitivity to docetaxel. **a** PC3 cells (1 × 10^4^/well) were co-cultured with MSCs in an existence of DTX (20 μM). The occurrence of autophagy was inhibited by autophagy inhibitor CQ (10 μM) or 3-MA (2 mM). CCK8 assay was used to examined the cell viability of PC3 cells. **b** The cell apoptosis of PC3 cells was analyzed by flow cytometry using Annexin V-FITC/PI. **c** CCK8 assay was used to examined the cell viability of DU145 cells. **d** The cell apoptosis of DU145 cells was analyzed by flow cytometry using Annexin V-FITC/PI. *P < 0.05; **P < 0.01

Furthermore, addition of autophagy inhibitors CQ or 3-MA to the cell culture was performed. The results showed that autophagy inhibitor could effectively restore the sensitivity of CRPC cell cocultured with MSCs to docetaxel, resulting in decrease of proliferation and increase of apoptosis in CRPC cells (Fig. 5). Our results suggest that MSCs promote CRPC cells docetaxel chemoresistance via inducing cell autophagy.

### MSCs induce CRPC cells autophagy and docetaxel chemoresistance depending on TGF-β1 secretion

**Fig. 6:**
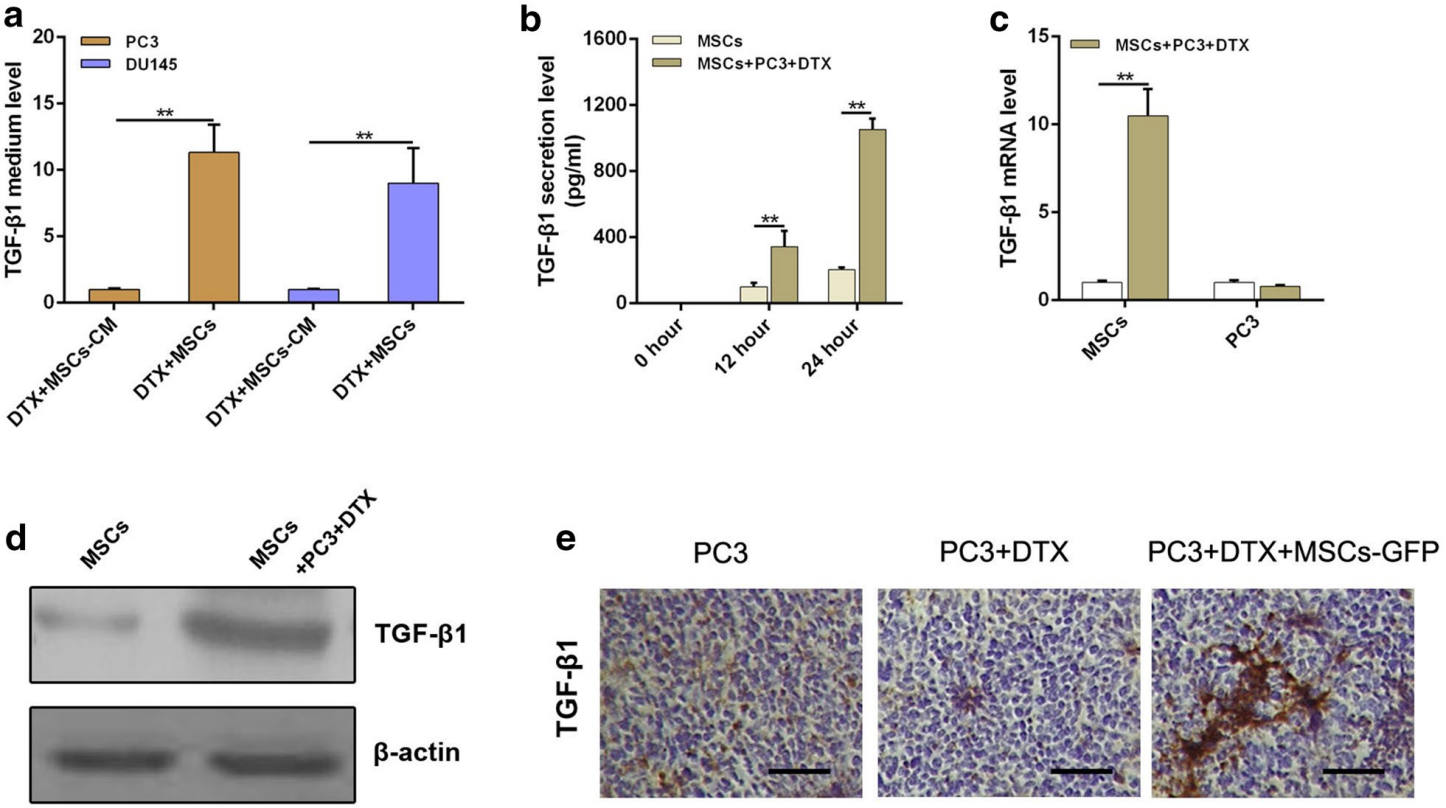
Docetaxel increases TGF-β1 secretion of MSCs when co-cultured with CRPC cells **a** ELISA assay was performed to examine the concentration of TGF-β1 in coculture medium of MSCs and PCa cells after DTX administration. **b** The amount of TGF-β1 in conditioned medium was detected by ELISA. **c** TGF-β1 mRNA in MSCs was assayed by real-time PCR. **d** The expression of TGF-β1 in protein level was examined by western blot. **e** Immunohistochemistry against TGF-β1 in tumour tissues was observed by microscope (original magnification ×200). **P < 0.01

It has been reported that TGF-β1 signaling plays a key role in regulation of autophagy [15]. So we tested TGF-β1 level in the conditioned medium (CM) obtained from CRPC cells treated with MSCs-CM or cocultured with MSCs in response to docetaxel administration. As is shown in Fig. 6a, TGF-β1 was dramatically increased in CM obtained from docetaxel administrated CRPC cells cocultured with MSCs. Additionally, TGF-β1 secretion was increased in time-dependent manner in MSCs when cocultured with docetaxel administrated PC3 cells (Fig. 6b). We then investigated the effect of docetaxel on secretion of TGF-β1 in MSCs and PC3 cells. Results showed that docetaxel could cause a significant up-regulation of TGF-β1 expression in MSCs, but not in PC3 cells, at both mRNA and protein levels (Fig. 6c and d). We also validated significantly increased TGF-β1 expression by immunohistochemistry in PC3 tumors with docetaxel and MSCs-GFP administration (Fig. 6e). These results indicate that MSCs cocultured with CRPC cells secret TGF-β1 increase when docetaxel administration performed.

**Fig. 7:**
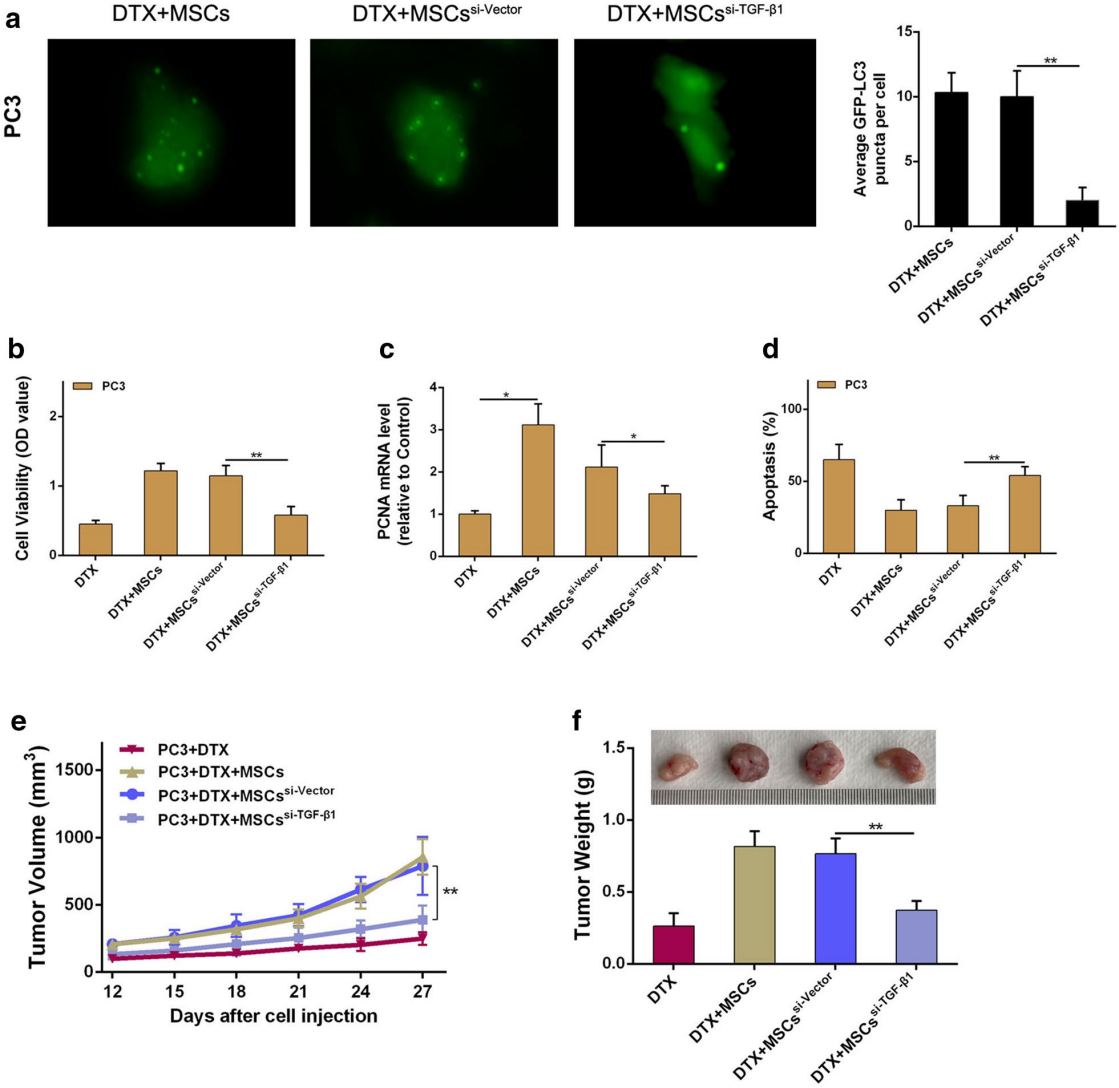
TGF-β1 is essential for MSCs induced CRPC cells autophagy and docetaxel chemoresistance. **a** MSCs (1 × 10^6^) growing to 50–60% confluence in 10 cm petri dishes were transfected with TGFβ1-targeting siRNA or their corresponding non-targeting siRNA using lentiviral vectors for 48 h. PC3 cells were transfected with GFP-tagged LC3. Images were taken under a fluorescence microscope. **b** PC3 cells (2 × 10^5^/well) were cocultured with MSCs in a 6-well plate with an existence of DTX for 48 h. CCK8 was employed to examine the proliferation of PC3 cells. **c** Real-time PCR was employed to examine the PCNA expression level of PC3 cells. Results were reported as ratio to control group. **d** PI/Annexin V-FITC assay was used to measure apoptotic PC3 cells by flow cytometry. **e** PC3 cells and MSCs were prepared either as single-cell type suspensions (1 × 10^6^ PC3 cells in 200 μL PBS) or a mix of cells (1 × 10^6^ PC3 cells and 2 × 10^5^ MSCs^si-TGF-β1^ in 200 μL PBS). PC3 cells (alone or mixed with MSCs^si-TGF-β1^) were subcutaneously administrated in the armpit area of nude mice with DTX administration. PC3 tumor volume was observed to evaluate the effect of TGF-β1 on tumor growth after DTX administration. **f** On 27 day after implantation, the animals were sacrificed. Tumor tissues were removed from mice and tumor weights were measured. *P < 0.05; **P < 0.01

To further verified the role of TGF-β1 in MSC-mediated CRPC docetaxel chemoresistance and cell autophagy, TGF-β1 was silenced in MSCs by siRNA. We found that the increased autophagy activation of docetaxel administrated PC3 cells upon MSCs coculture was obviously suppressed when MSCs suffered TGF-β1 knockdown (Fig. 7a). Effects of si-TGF-β1 on blocking MSCs-induced PC3 cells proliferation increase and apoptosis inhibition were also demonstrated (Fig. 7b–d). In addition, results in xenotransplant tumor model showed that TGF-β1 knockdown significantly inhibited the effect of MSCs on increasing the volume of prostate tumor in docetaxel administration (Fig. 7e, f). Combined with above, TGF-β1 is essential for MSCs-induced CRPC cells autophagy and docetaxel chemoresistance.

## Discussion

Docetaxel chemoresistance remains an important obstacle to the cure of metastatic CRPC today. Stroma cells in tumor microenvironment were recognized as important contributors in facilitating the development of chemotherapy resistance [16]. MSCs, also called multipotent mesenchymal stromal cells, are a heterogeneous subset of stromal stem cells contributing to tissue homeostasis and regeneration. Previous studies have reported that MSCs can promote cisplatin chemotherapy resistance by secreting protective cytokines to help cancer cells overcome the anticancer effect of chemotherapeutic agents [17]. In current study, we evaluated the role of MSCs in the development of CRPC cells chemoresistance to docetaxel. We found that MSCs desensitize CRPC cells to docetaxel and accelerate chemoresistance to docetaxel both in vivo and in vitro experiments (Figs. 1 and 3). Further study showed that MSCs could alleviate docetaxel-induced CRPC cell proliferation inhibition and apoptosis increase (Fig. 2).

Autophagy has been reported to perform critical roles for cell survival in response to stress [18–20]. Autophagic degradation acts as a key source of fatty acids, amino acids, nucleotides, and other precursor molecules maintaining cellular homeostasis and facilitating cell survival. Recently, growing evidence demonstrates that autophagy is associated with various pathological and physiological processes, including tumorigenesis, chemoresistance, cell differentiation and adaptation to changed environmental conditions [21, 22]. Autophagy also promotes tumor cell proliferation, angiogenesis and metastasis, accelerating cancer progression. Song et al. showed that autophagy could decrease the sensitivity of hepatoma cells to chemotherapeutic agents by affecting their apoptotic potential [23]. In present study, we performed studies in PC3 and DU145 cells, confirming that MSCs could significantly induce autophagy in CRPC cells (Fig. 4). In addition, we have shown that inhibition of autophagy could restore CRPC cell sensitivity to docetaxel (Fig. 5), suggesting that MSCs-induced cell autophagy serves as a protective mechanism for CRPC cells to resist the cell toxicity of docetaxel.

It is reported that MSCs communicate with cancer cells mostly by secreting soluble factors. Besides that, MSCs could secrete multiple cytokines when exposure to various local microenvironment. TGF-β1, as a key cytokine secreted by MSCs, is related to various tumor cell invasion and migration by regulating some cytokines expression. TGF-β1 also plays an important role in tumor cell autophagy activation [15]. In present study, we found that TGF-β1 was dramatically increased in CM obtained from docetaxel administrated CRPC cells cocultured with MSCs. Further study found that TGF-β1 secretion in MSCs increased in time-dependent manner when cocultured with PC3 cells under docetaxel administration (Fig. 6). Inhibiting MSCs secreting TGF-β1 diminished the ability of MSCs in inducing cell autophagy and docetaxel chemoresistance in CRPC, indicating that TGF-β1 is essential for MSCs induced CRPC cell autophagy and docetaxel chemoresistance. Meanwhile, we found that MSCs secreting TGF-β1 would increase when cocultured with docetaxel administrated PC3 cells (Fig. 6b, c). However, detail mechanisms about the difference of TGF-β1 secretion need a further investigation. We also found that MSCs and their conditioned medium were significantly different in improving CRPC cell proliferation and reducing cell apoptosis after docetaxel treatment. As we know, MSCs as an important mediator in TME play important roles in tumor progression via secreting various cytokines as well as direct contact with adjacent cancer cells. In our previous study, we also found that MSCs in mixed co-culture system showed a more enhancement of PC3 cell stemness than that in transwell-culture system, which implies that MSCs can accelerate PCa growth through their secretory effects, as well as in a cell–cell contact manner. Therefore, the difference between MSCs and their conditioned medium in improving CRPC cell proliferation and reducing cell apoptosis after docetaxel treatment would be reasonable.

In conclusion, our results revealed that MSCs could desensitize CRPC to docetaxel chemotherapy and accelerate chemoresistance occurrence via secreting TGF-β1 and inducing cell autophagy. The results suggest that docetaxel treatment in clinical PCa therapy may elicit the expression of TGF-β1 in MSCs, which will result in docetaxel chemoresistance occurrence. We expect that our findings will offer insights into further explore on the mechanism of prostate cancer development and provide a theoretical basis for finding new therapies for prostate cancer.

## Data Availability

Please contact the corresponding author for data requests.

## Abbreviations

PCa: Prostate cancer
MSCs: Mesenchymal stem cells
ADT: Androgen deprivation therapy
CRPC: Castration-resistant prostate cancer
TME: Tumor microenvironment
FBS: Fetal bovine serum
GFP: Green fluorescent protein
RT-PCR: Real-time quantitative PCR
siRNA: Small interfering RNA
ELISA: Enzyme linked immunosorbent assay
GFP: Green fluorescent protein
LC3: Microtubule-associated protein 1 light chain 3
DTX: Docetaxel

## References

[1] Siegel RL, Miller KD, Jemal A (2020). Cancer statistics, 2020. CA Cancer J Clin.

[2] Chen W, Zheng R, Baade PD, Zhang S, Zeng H, Bray F, Jemal A, Yu XQ, He J (2016). Cancer statistics in China, 2015. CA Cancer J Clin.

[3] Karantanos T, Evans CP, Tombal B, Thompson TC, Montironi R, Isaacs WB (2015). Understanding the mechanisms of androgen deprivation resistance in prostate cancer at the molecular level. Eur Urol.

[4] Francini E, Sweeney CJ (2016). Docetaxel activity in the era of life-prolonging hormonal therapies for metastatic castration-resistant prostate cancer. Eur Urol.

[5] Jia J, Zhang HB, Shi Q, Yang C, Ma JB, Jin B, Wang X, He D, Guo P (2019). KLF5 downregulation desensitizes castration-resistant prostate cancer cells to docetaxel by increasing BECN1 expression and inducing cell autophagy. Theranostics.

[6] Yeldag G, Rice A, Del Rio Hernandez A (2018). Chemoresistance and the self-maintaining tumor microenvironment. Cancers (Basel)..

[7] Deans RJ, Moseley AB (2000). Mesenchymal stem cells: biology and potential clinical uses. Exp Hematol.

[8] Karnoub AE, Dash AB, Vo AP, Sullivan A, Brooks MW, Bell GW, Richardson AL, Polyak K, Tubo R, Weinberg RA (2007). Mesenchymal stem cells within tumour stroma promote breast cancer metastasis. Nature.

[9] Luo J, Ok Lee S, Liang L, Huang CK, Li L, Wen S, Chang C (2014). Infiltrating bone marrow mesenchymal stem cells increase prostate cancer stem cell population and metastatic ability via secreting cytokines to suppress androgen receptor signaling. Oncogene.

[10] Yu Y, Liu Y, Zong C, Yu Q, Yang X, Liang L, Ye F, Nong L, Jia Y, Lu Y, Han Z (2016). Mesenchymal stem cells with Sirt1 overexpression suppress breast tumor growth via chemokine-dependent natural killer cells recruitment. Sci Rep.

[11] Jing Y, Han Z, Liu Y, Sun K, Zhang S, Jiang G, Li R, Gao L, Zhao X, Wu D (2012). Mesenchymal stem cells in inflammation microenvironment accelerates hepatocellular carcinoma metastasis by inducing epithelial-mesenchymal transition. PLoS One.

[12] Cheng J, Yang K, Zhang Q, Yu Y, Meng Q, Mo N, Zhou Y, Yi X, Ma C, Lei A, Liu Y (2016). The role of mesenchymal stem cells in promoting the transformation of androgen-dependent human prostate cancer cells into androgen-independent manner. Sci Rep.

[13] Yu Y, Zhang Q, Ma C, Yang X, Lin R, Zhang H, Liu Y, Han Z, Cheng J (2018). Mesenchymal stem cells recruited by castration-induced inflammation activation accelerate prostate cancer hormone resistance via chemokine ligand 5 secretion. Stem Cell Res Ther.

[14] Lund K, Olsen CE, Wong JJW, Olsen PA, Solberg NT, Hogset A, Krauss S, Selbo PK (2017). 5-FU resistant EMT-like pancreatic cancer cells are hypersensitive to photochemical internalization of the novel endoglin-targeting immunotoxin CD105-saporin. J Exp Clin Cancer Res.

[15] Suzuki HI, Kiyono K, Miyazono K (2010). Regulation of autophagy by transforming growth factor-beta (TGF-beta) signaling. Autophagy.

[16] Howard N, Clementino M, Kim D, Wang L, Verma A, Shi X, Zhang Z, DiPaola RS (2019). New developments in mechanisms of prostate cancer progression. Semin Cancer Biol.

[17] Roodhart JM, Daenen LG, Stigter EC, Prins HJ, Gerrits J, Houthuijzen JM, Gerritsen MG, Schipper HS, Backer MJ, van Amersfoort M (2011). Mesenchymal stem cells induce resistance to chemotherapy through the release of platinum-induced fatty acids. Cancer Cell.

[18] Mizushima N, Levine B, Cuervo AM, Klionsky DJ (2008). Autophagy fights disease through cellular self-digestion. Nature.

[19] Ding WX, Yin XM (2012). Mitophagy: mechanisms, pathophysiological roles, and analysis. Biol Chem.

[20] Mizushima N, Yoshimori T, Levine B (2010). Methods in mammalian autophagy research. Cell.

[21] Hu F, Zhao Y, Yu Y, Fang JM, Cui R, Liu ZQ, Guo XL, Xu Q (2018). Docetaxel-mediated autophagy promotes chemoresistance in castration-resistant prostate cancer cells by inhibiting STAT3. Cancer Lett.

[22] Sui X, Chen R, Wang Z, Huang Z, Kong N, Zhang M, Han W, Lou F, Yang J, Zhang Q (2013). Autophagy and chemotherapy resistance: a promising therapeutic target for cancer treatment. Cell Death Dis.

[23] Song J, Qu Z, Guo X, Zhao Q, Zhao X, Gao L, Sun K, Shen F, Wu M, Wei L (2009). Hypoxia-induced autophagy contributes to the chemoresistance of hepatocellular carcinoma cells. Autophagy.

